# Supplementary material for: Comparison of Tepotinib, Paclitaxel, or Ramucirumab Efficacy According to the Copy Number or Phosphorylation Status of the MET Gene: Doublet Treatment versus Single Agent Treatment
Source: Int J Mol Sci. 2024 Feb 1;25(3):1769. doi: 10.3390/ijms25031769 (PMC10855451; doi:10.3390/ijms25031769)

FACs data

Cell lines SNU620, MKN45, HS746T, SNU638, AGS

• SNU620

• **Control**

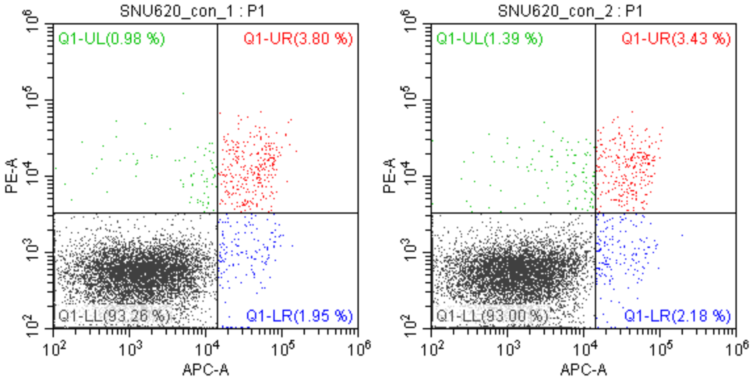

• **Ramucirumab 10nM**

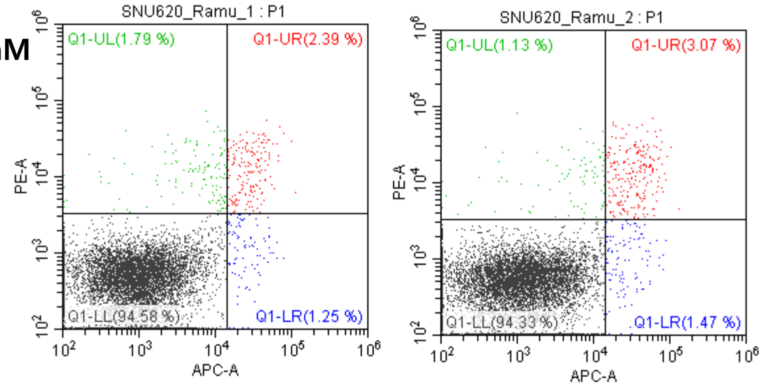

• **PTX 20nM**

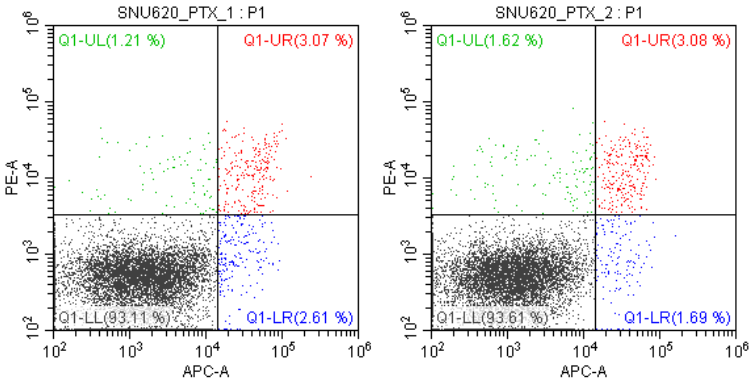

• **Tepotinib 10nM**

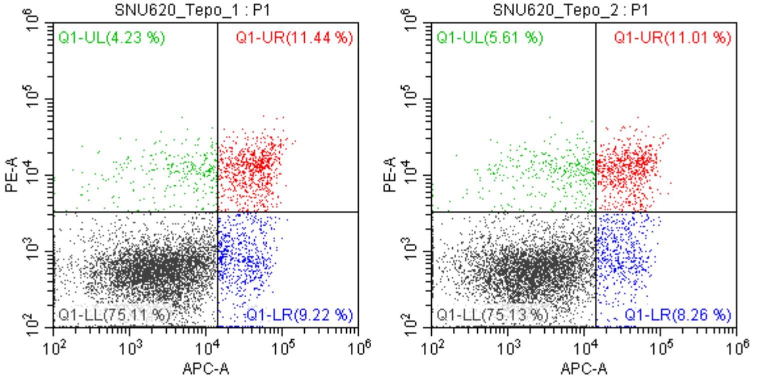

• **Tepotinib 10nM+PTX20nM**

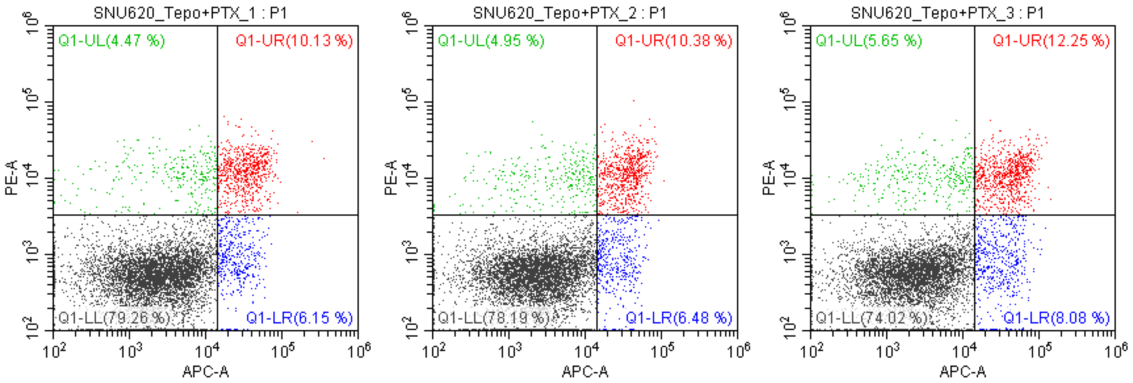

• **Ramucirumab 10nM+PTX20nM**

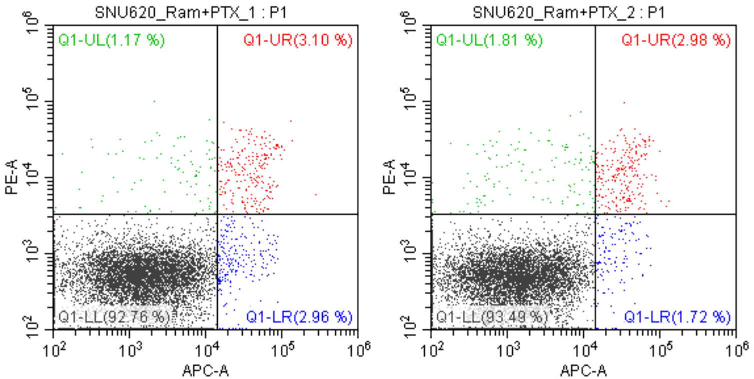

SNU620

- Control

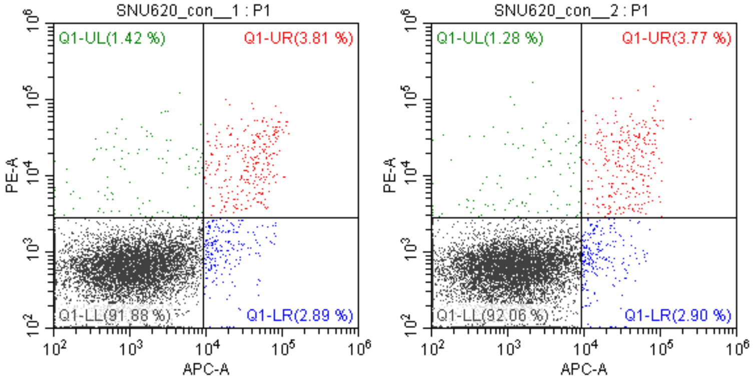

- Ramucirumab 10nM

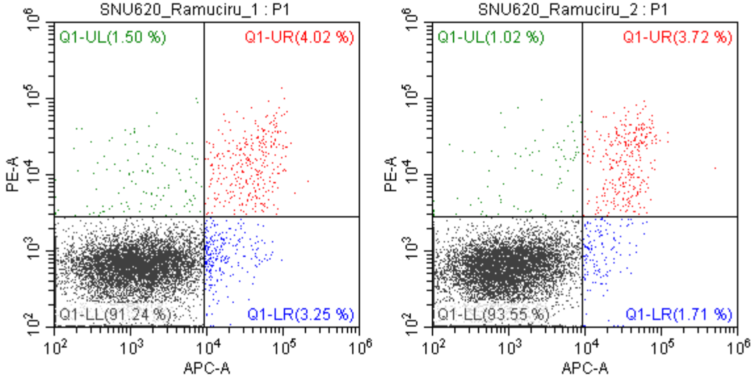

- PTX 20nM

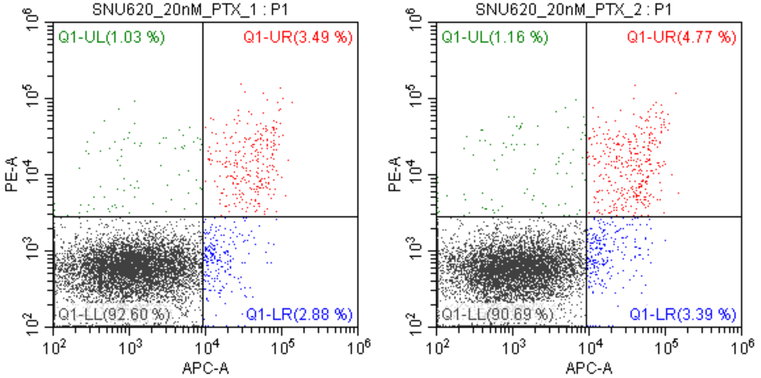

- Tepotinib 10nM

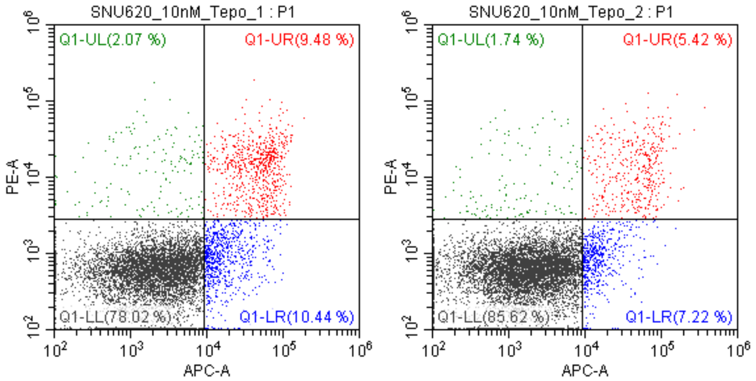

- Tepotinib 10nM+PTX20nM

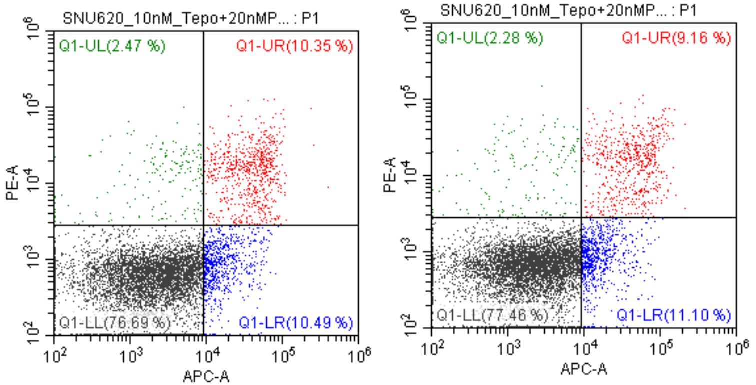

- Ramucirumab 10nM+PTX20nM

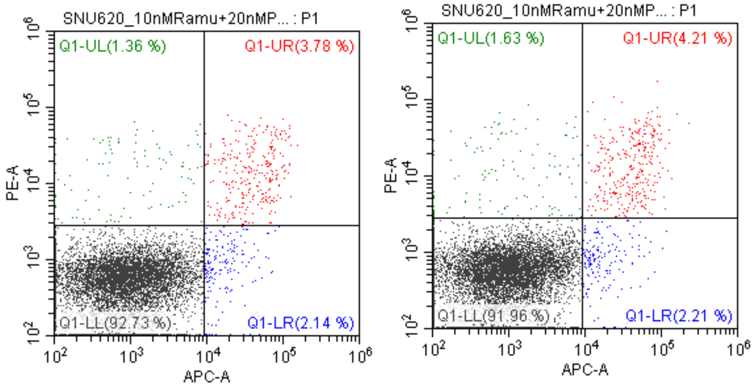

- **SNU620**

  - **Control**

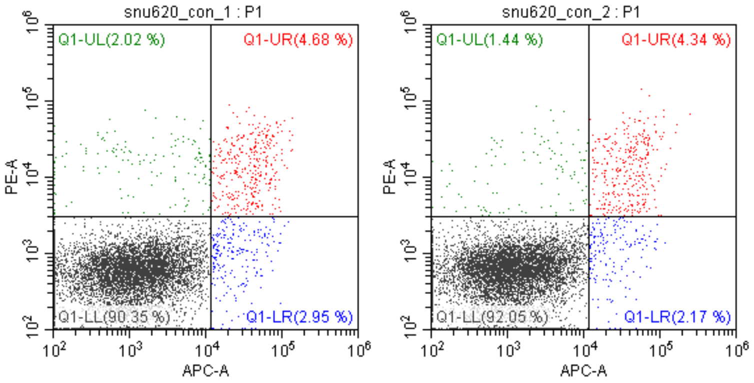

  - **Ramucirumab 10nM**

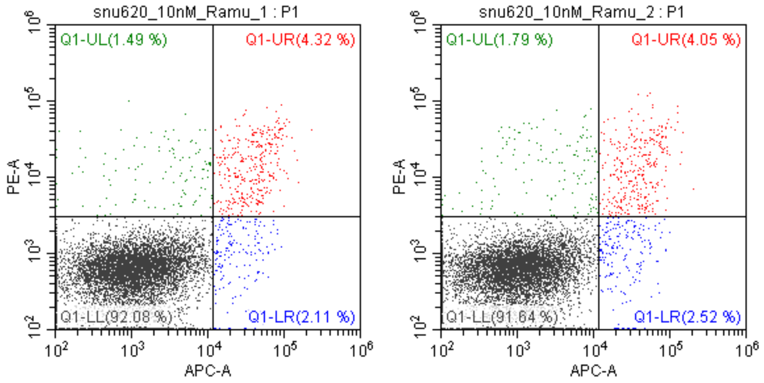

  - **PTX 20nM**

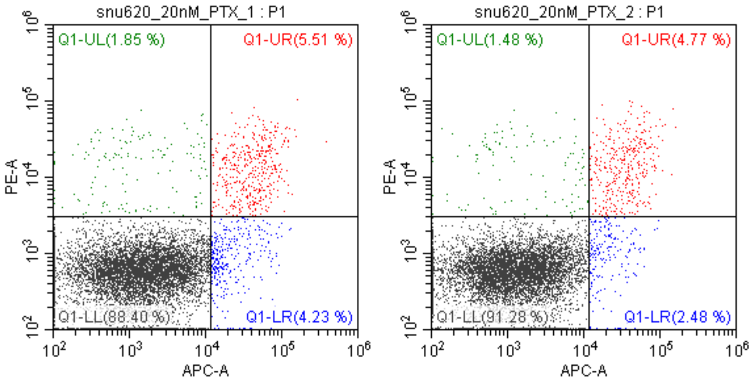

  - **Tepotinib 10nM**

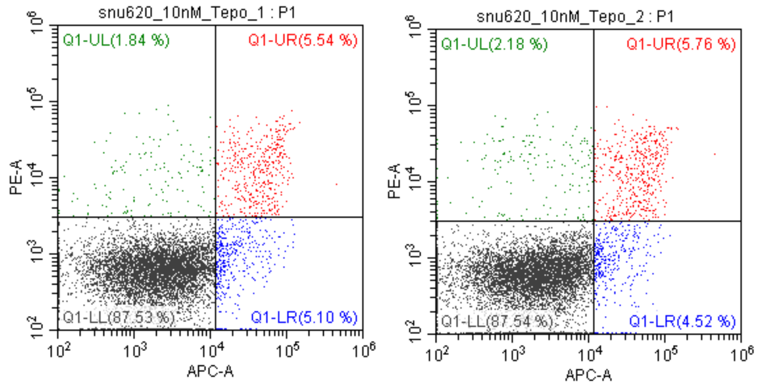

  - **Tepotinib 10nM+PTX20nM**

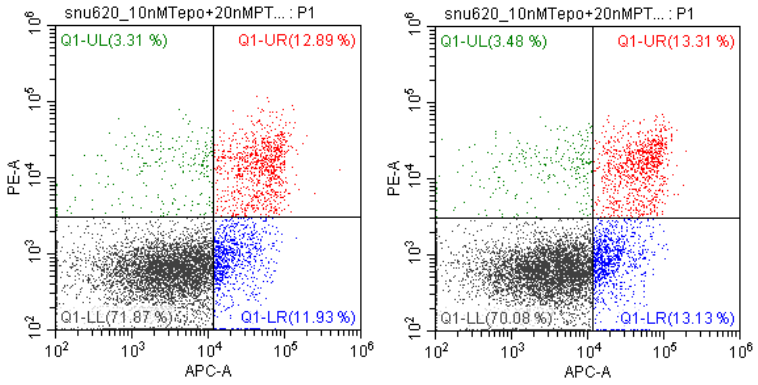

  - **Ramucirumab 10nM+PTX20nM**

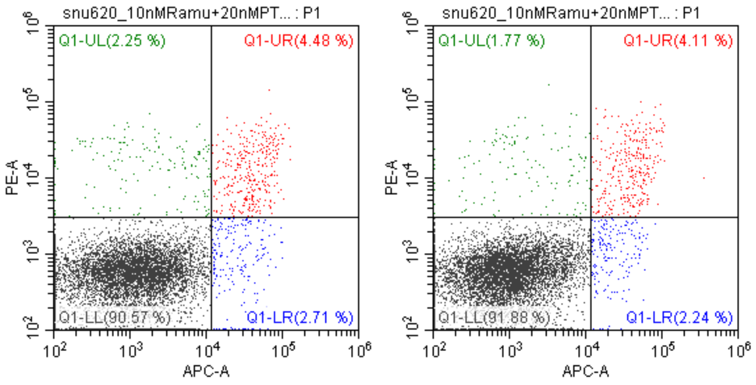

◆ MKN45

- **Control**

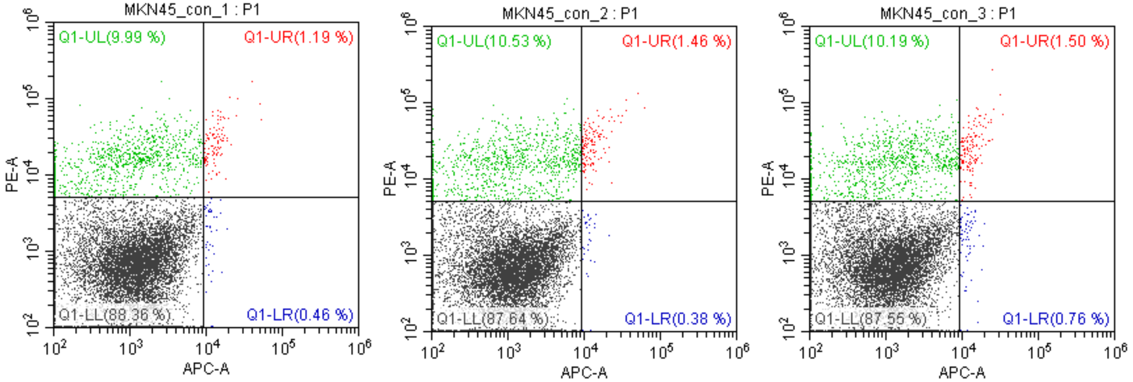

- **Ramucirumab 10nM**

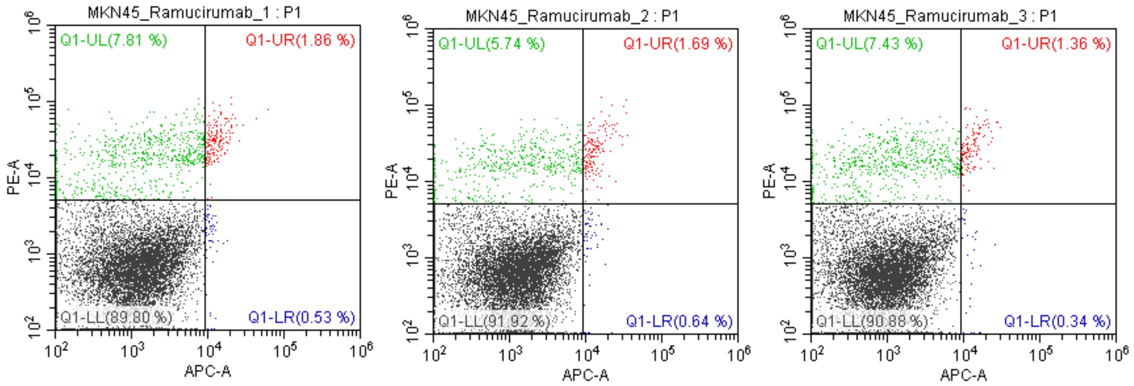

- **PTX 20nM**

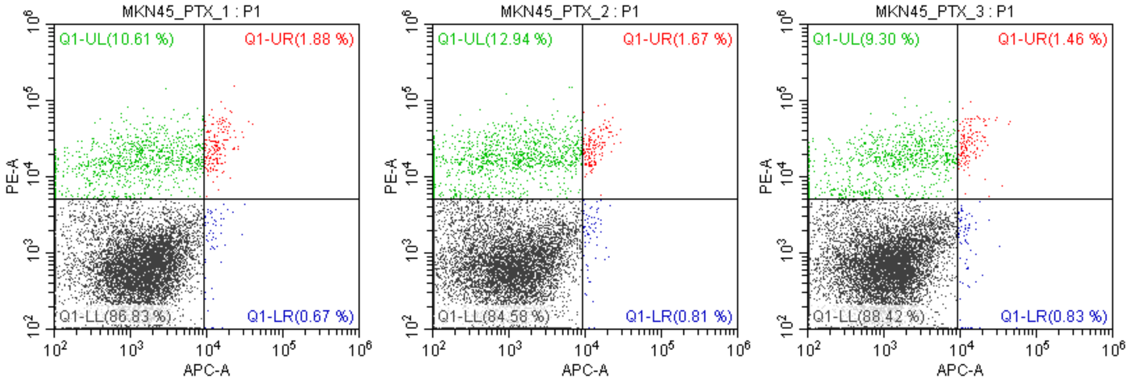

- **Tepotinib 10nM**

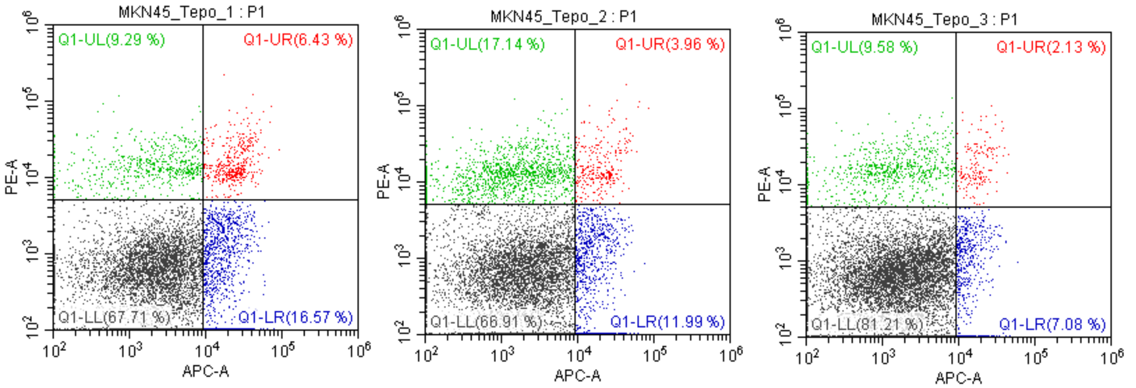

- **Tepotinib 10nM+PTX20nM**

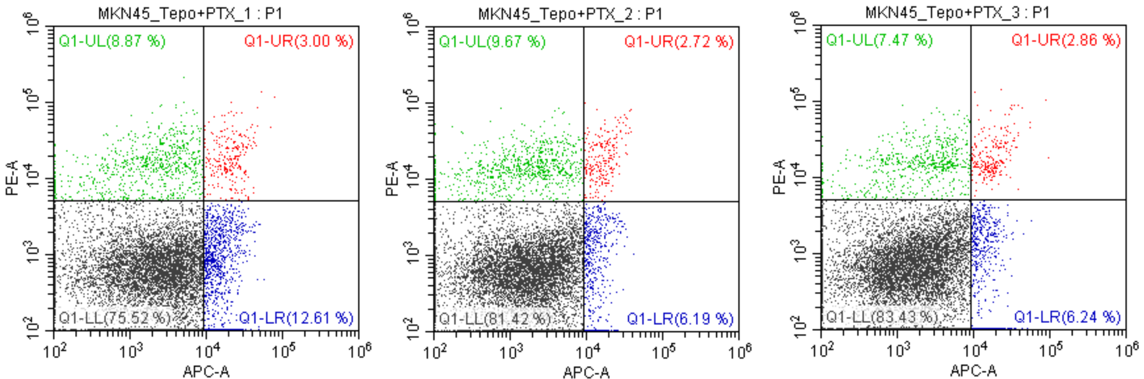

- **Ramucirumab 10nM+PTX20nM**

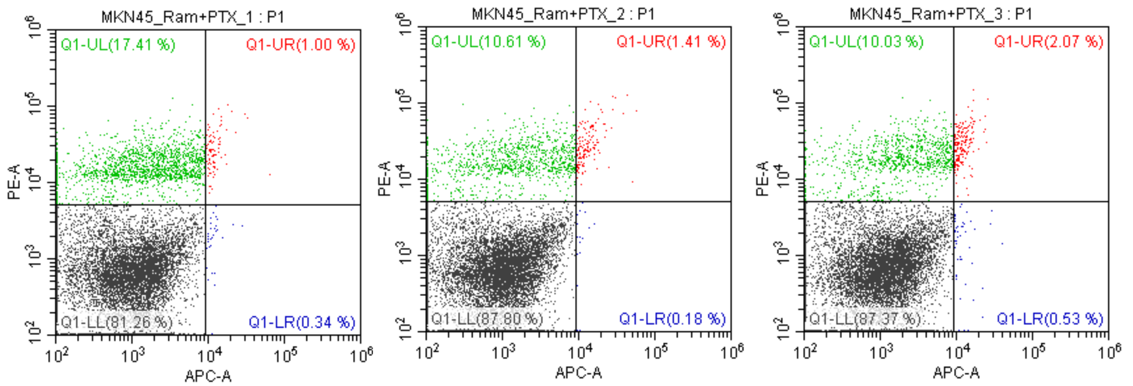

- **MKN45**

- **Control**

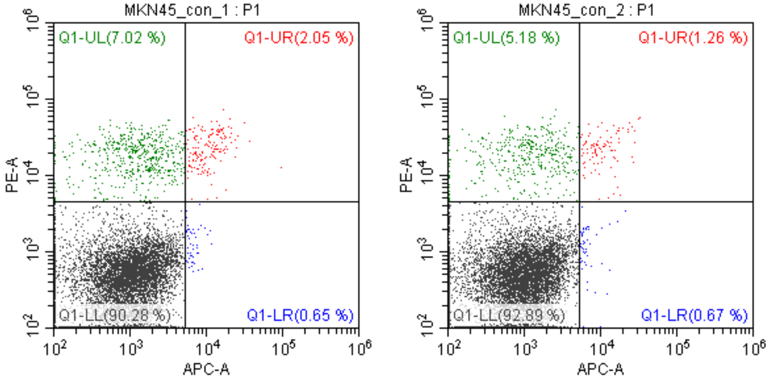

- **Ramucirumab 10nM**

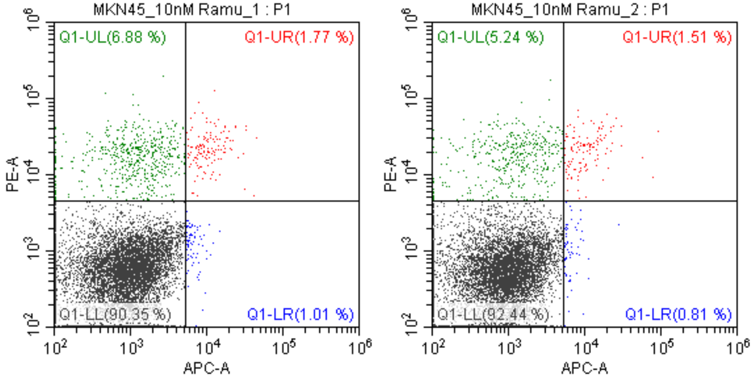

- **PTX 20nM**

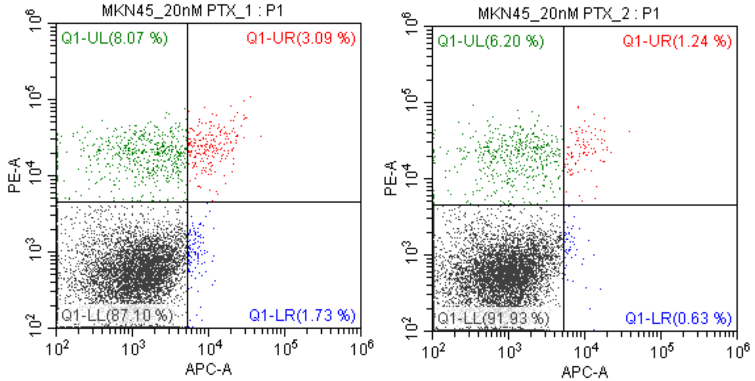

- **Tepotinib 10nM**

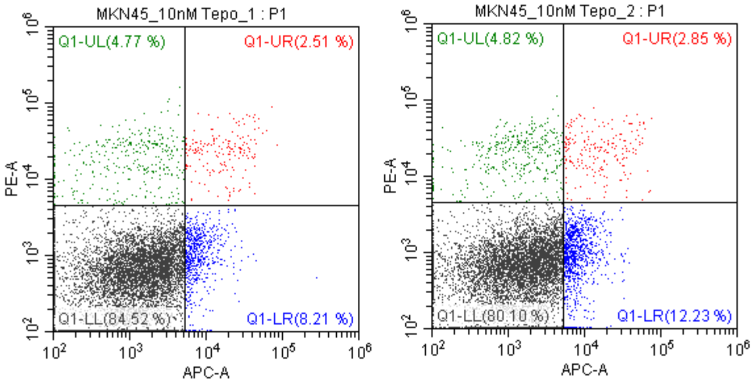

- **Tepotinib 10nM+PTX20nM**

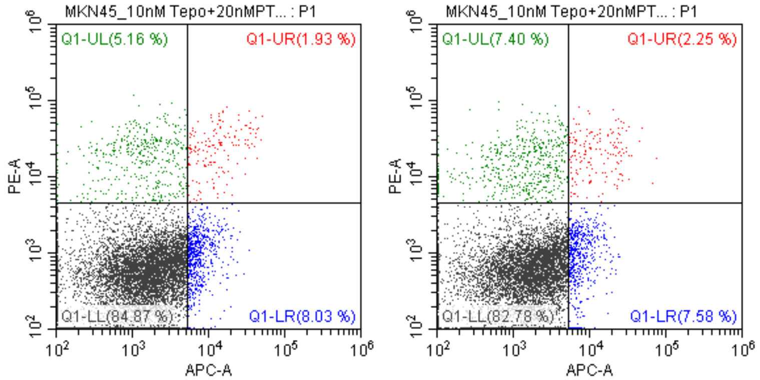

- **Ramucirumab 10nM+PTX20nM**

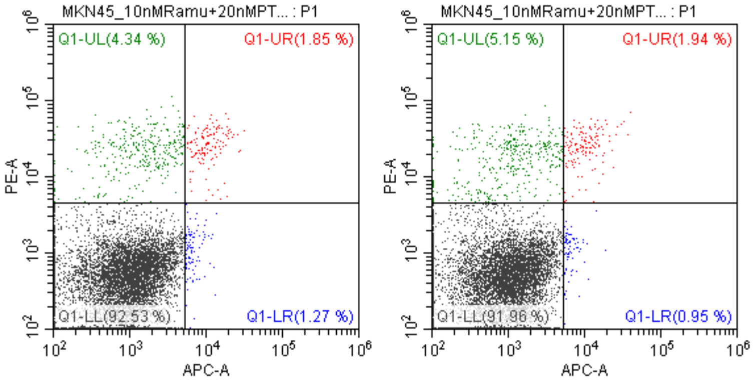

MKN45

- **Control**

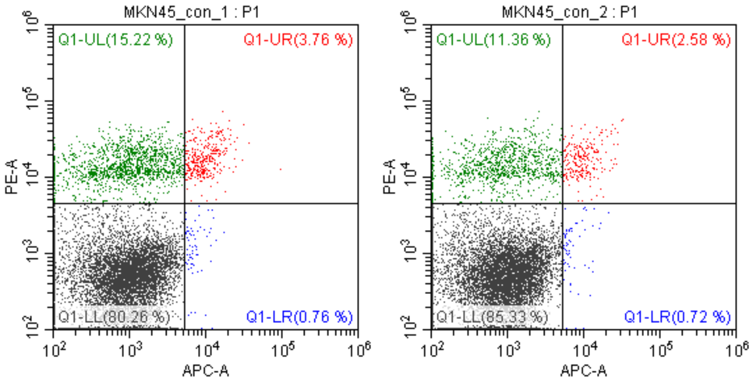

- **Ramucirumab 10nM**

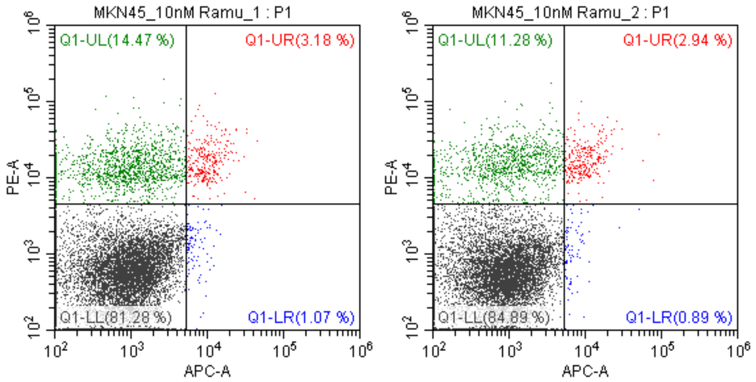

- **PTX 20nM**

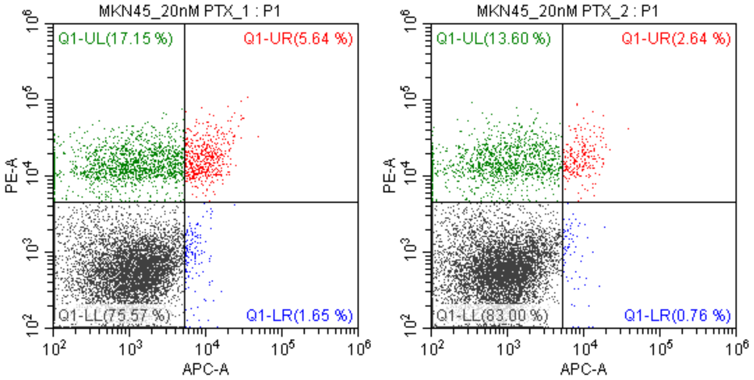

- **Tepotinib 10nM**

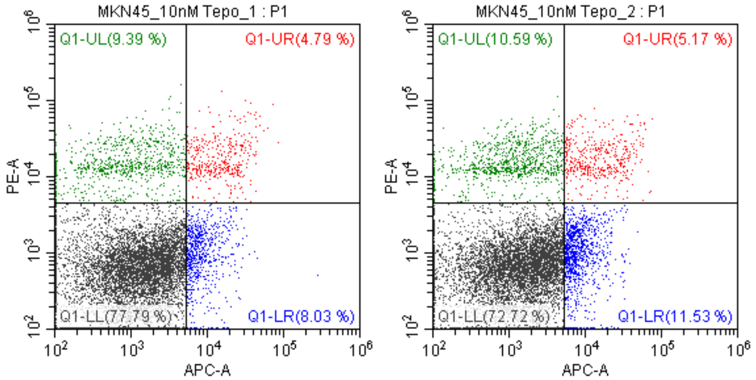

- **Tepotinib 10nM+PTX20nM**

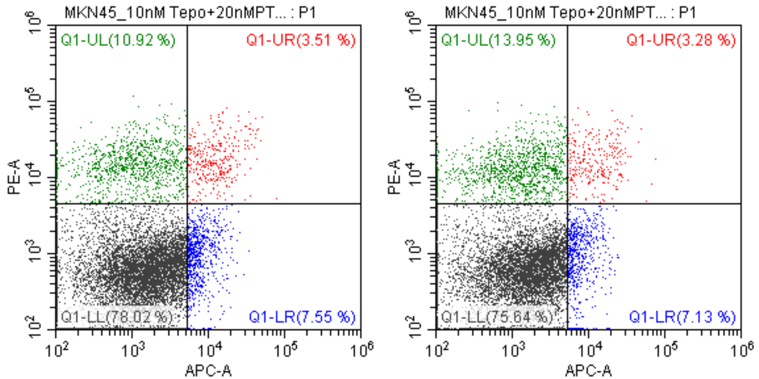

- **Ramucirumab 10nM+PTX20nM**

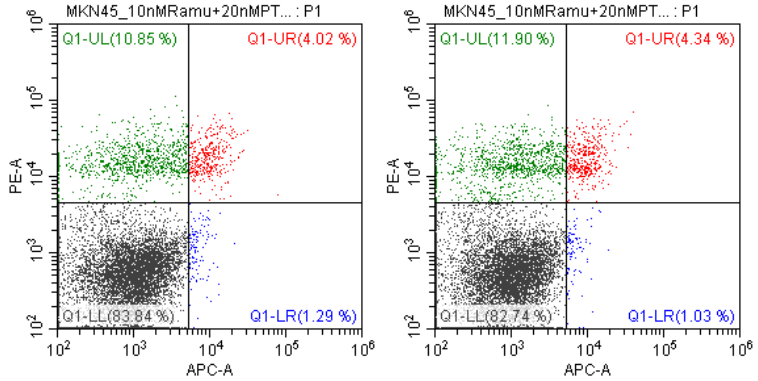

• HS746T

• Control

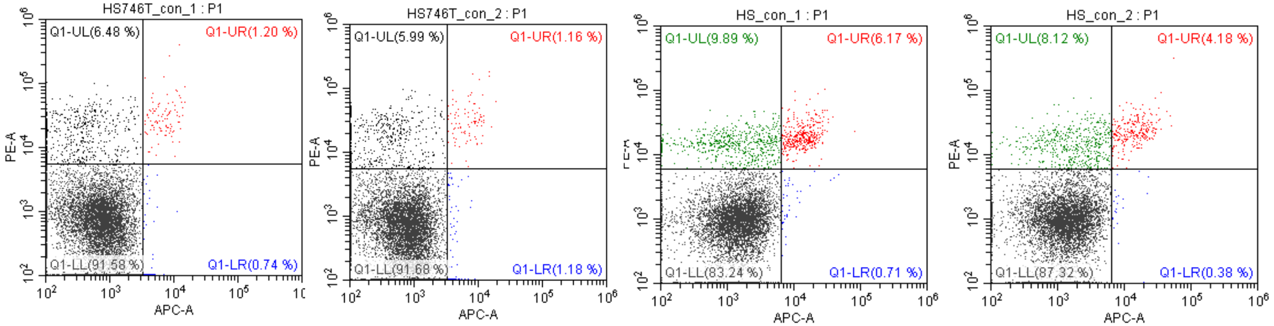

• Ramucirumab 10nM

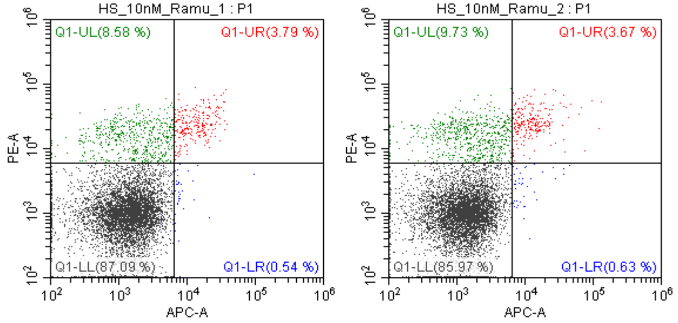

• Tepotinib 10nM

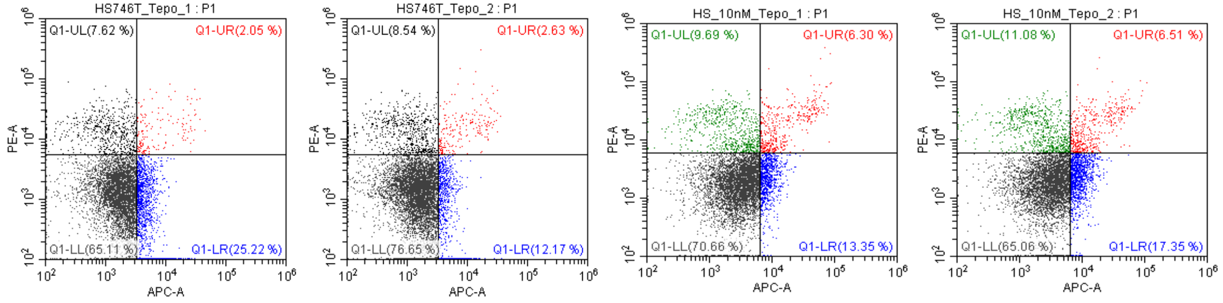

• PTX 20nM

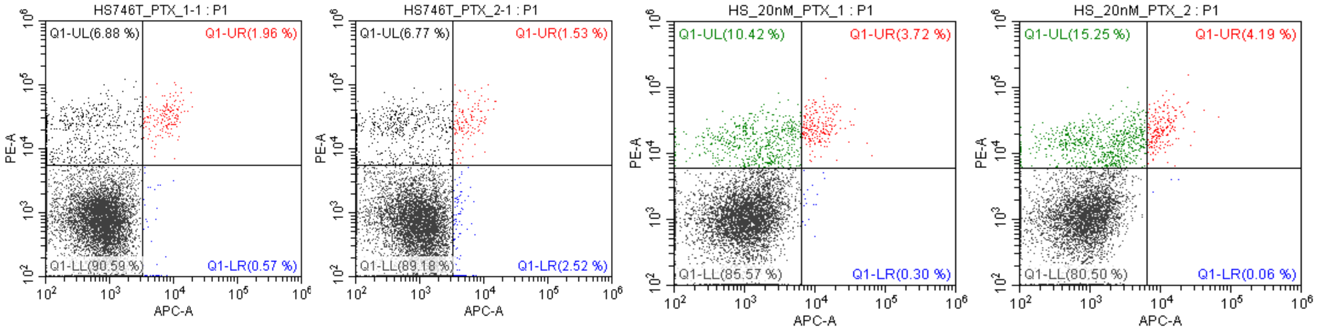

• Ramucirumab 10nM+PTX20nM

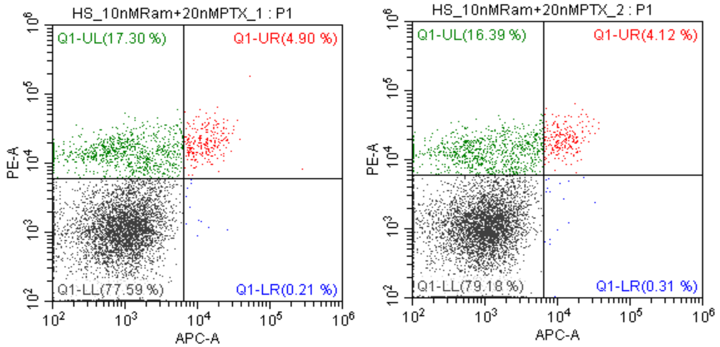

• Tepotinib 10nM+PTX20nM

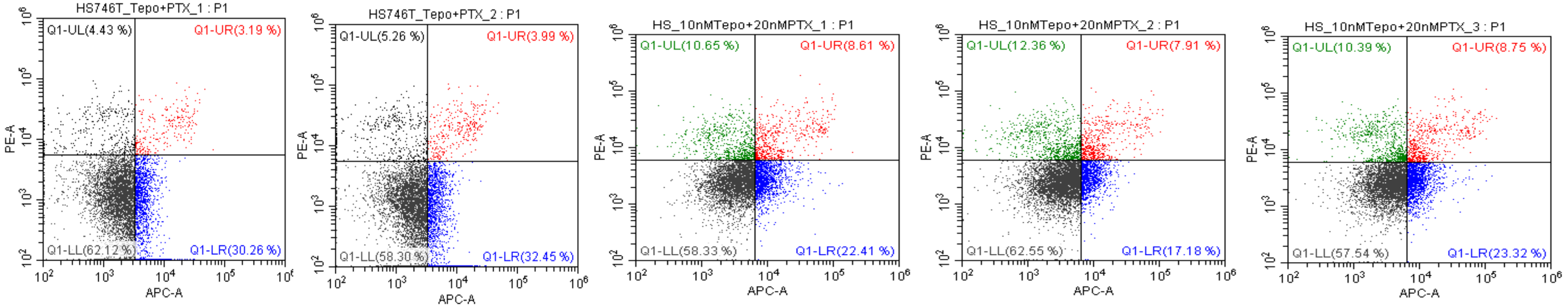

• SNU638

• **Control**

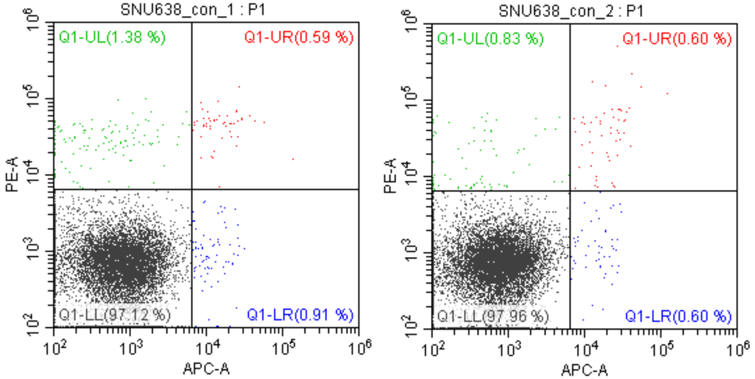

• **Ramucirumab 10nM**

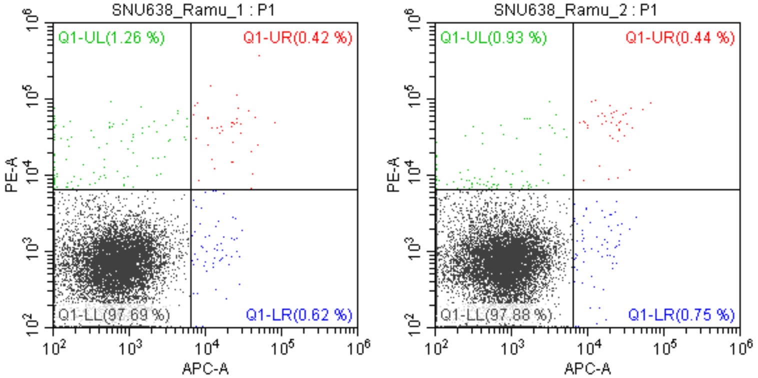

• **PTX 20nM**

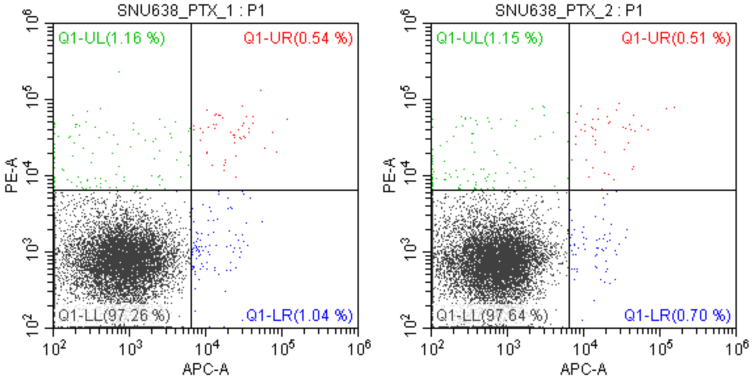

• **Tepotinib 10nM**

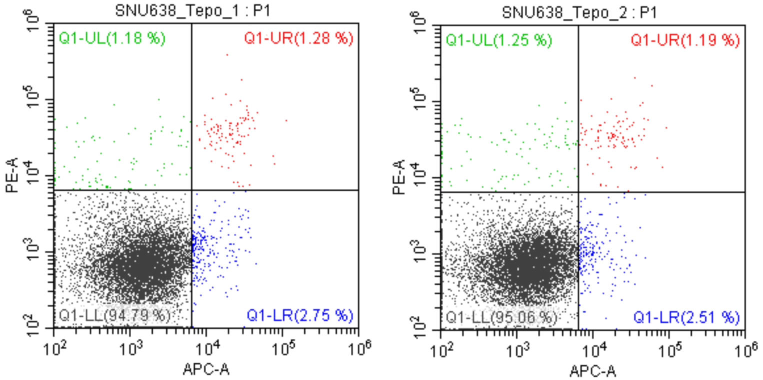

• **Tepotinib 10nM+PTX20nM**

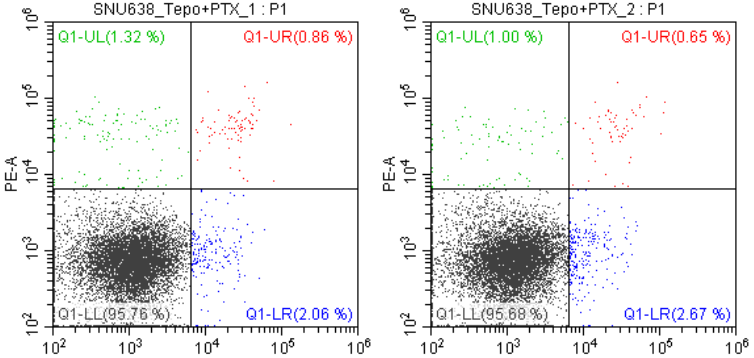

• **Ramucirumab 10nM+PTX20nM**

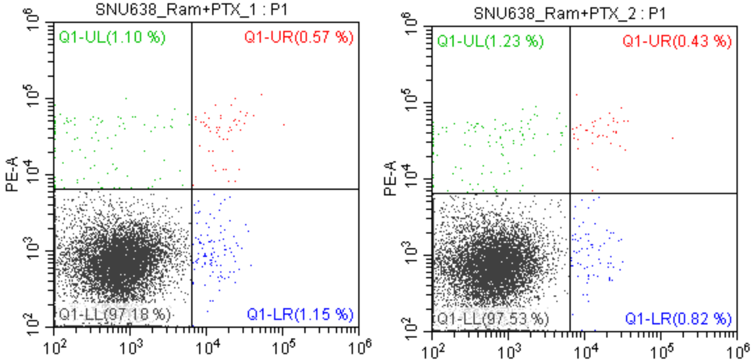

◆ SNU638

- **Control**

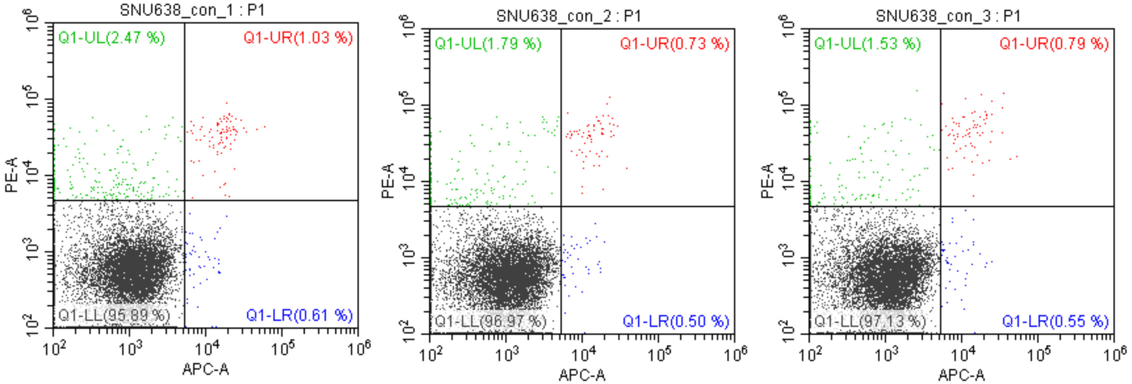

- **Ramucirumab 10nM**

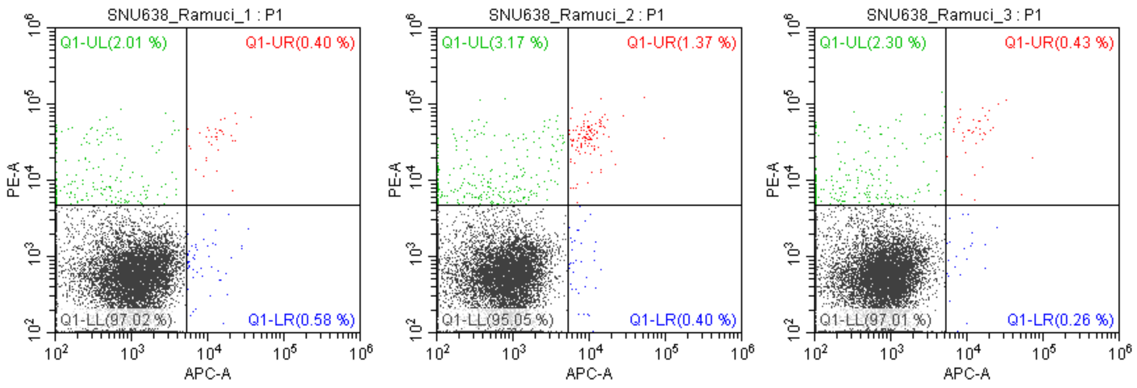

- **PTX 20nM**

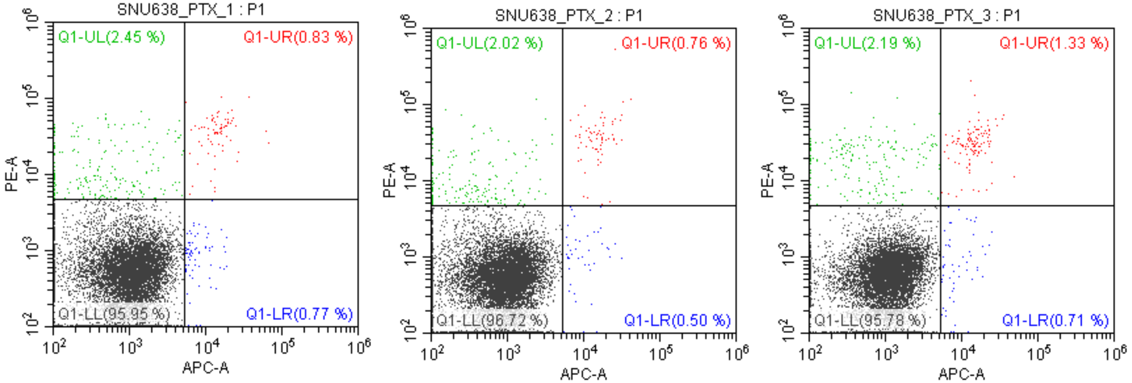

- **Tepotinib 10nM**

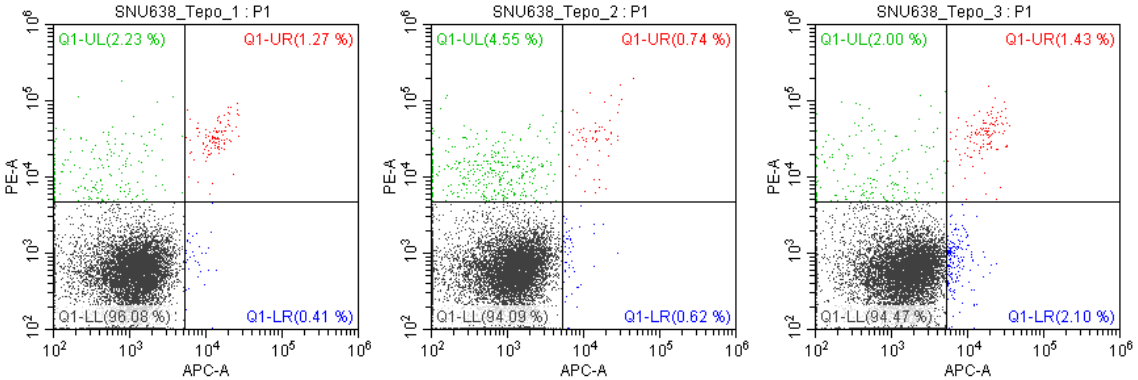

- **Tepotinib 10nM+PTX20nM**

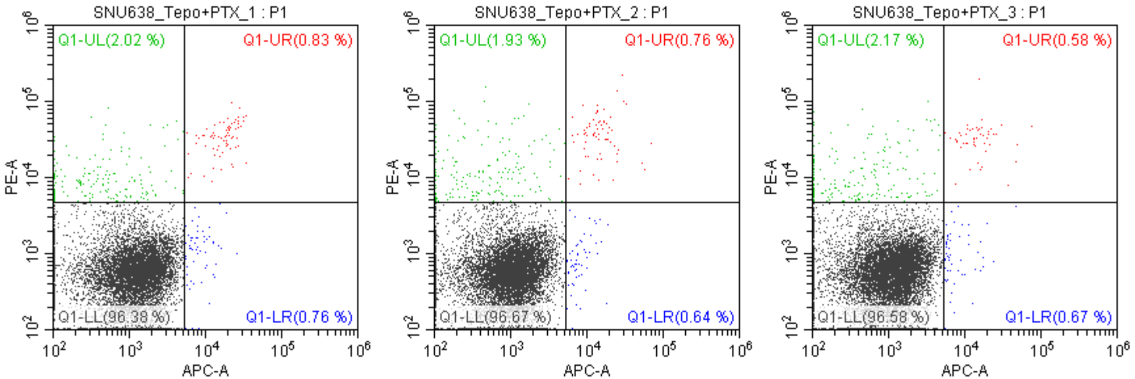

- **Ramucirumab 10nM+PTX20nM**

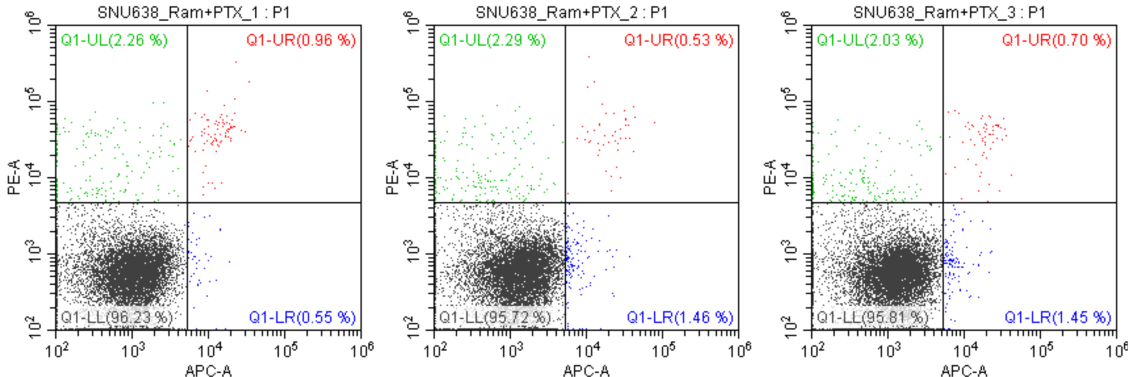

- **AGS**

- **Control**

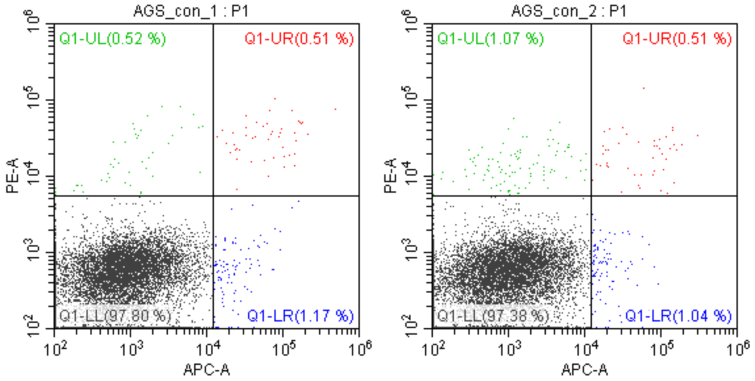

- **Ramucirumab 10nM**

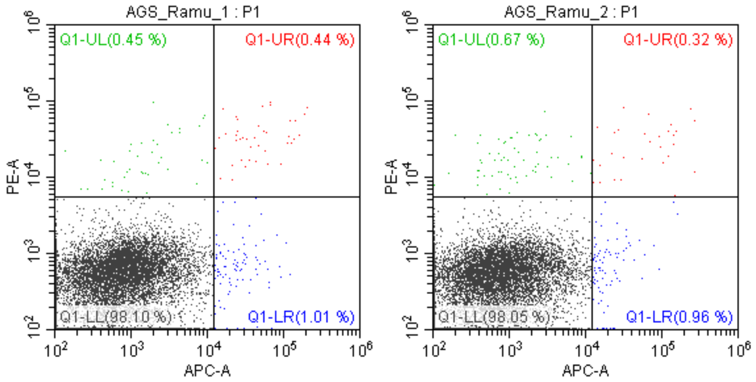

- **PTX 20nM**

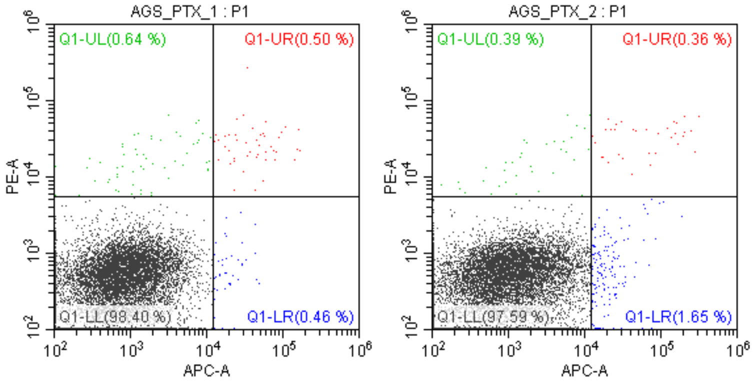

- **Tepotinib 10nM**

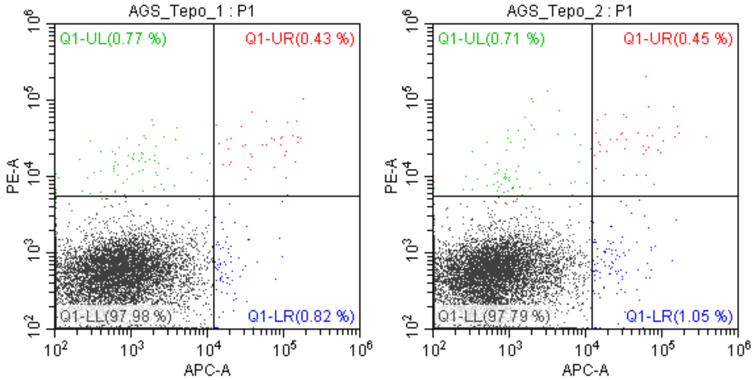

- **Tepotinib 10nM+PTX20nM**

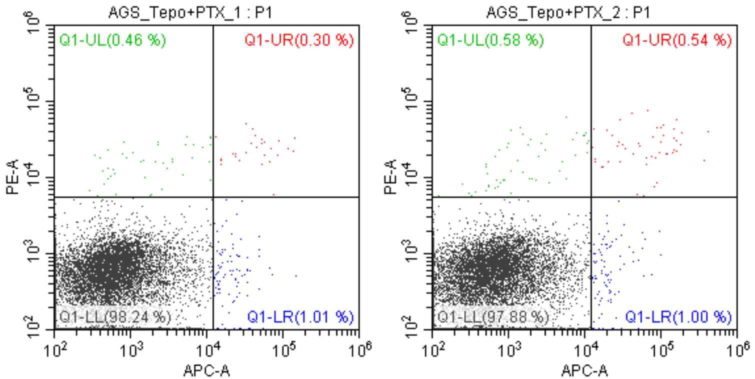

- **Ramucirumab 10nM+PTX20nM**

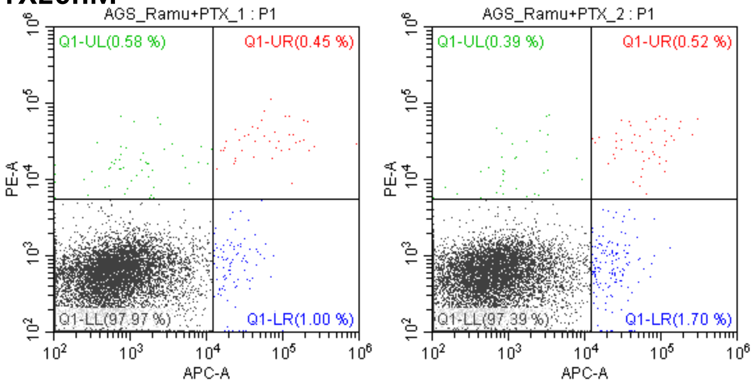

◆ AGS

- **Control**

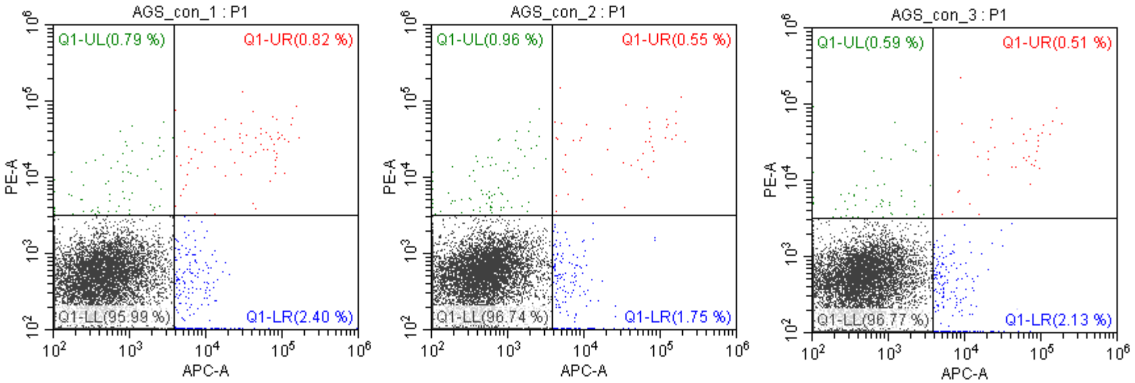

- **Ramucirumab 10nM**

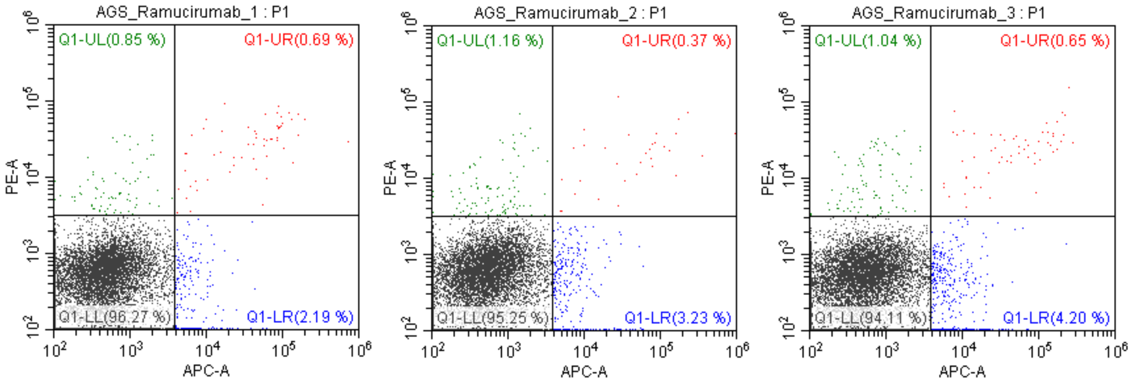

- **PTX 20nM**

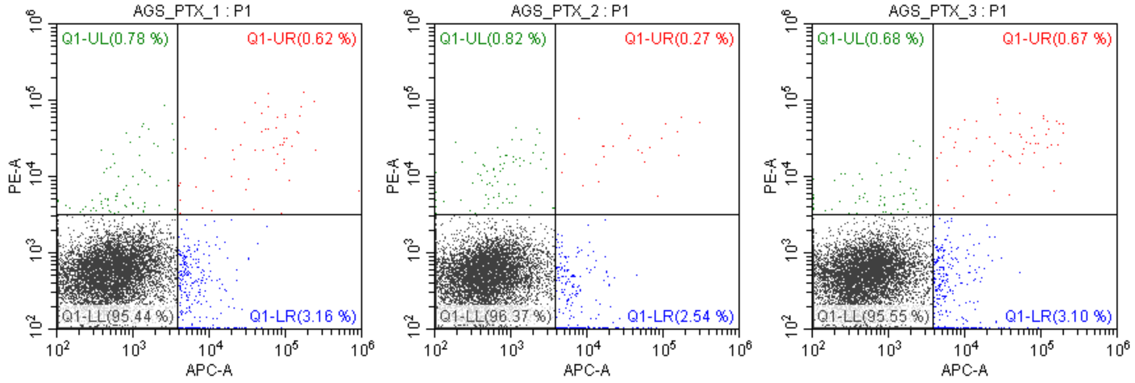

- **Tepotinib 10nM**

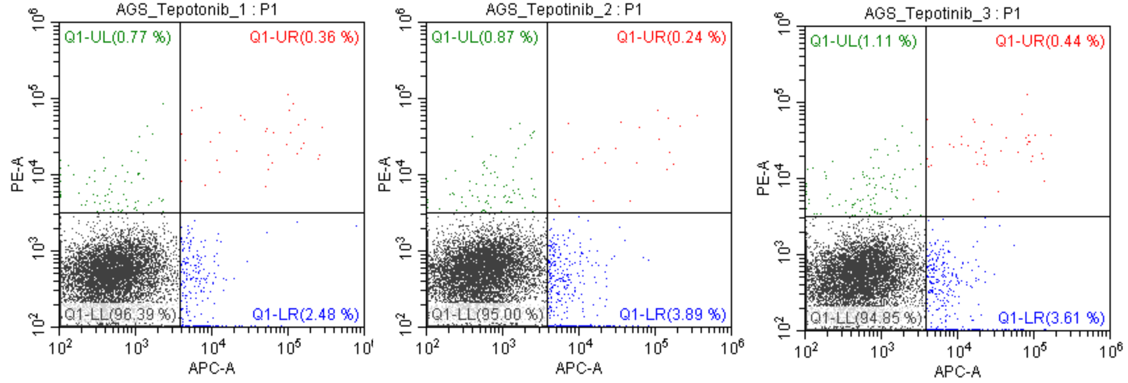

- **Tepotinib 10nM+PTX20nM**

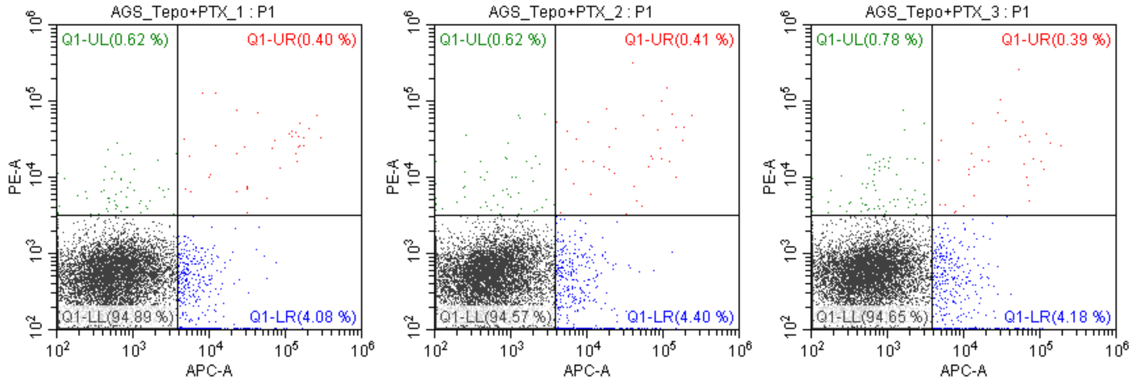

- **Ramucirumab 10nM+PTX20nM**

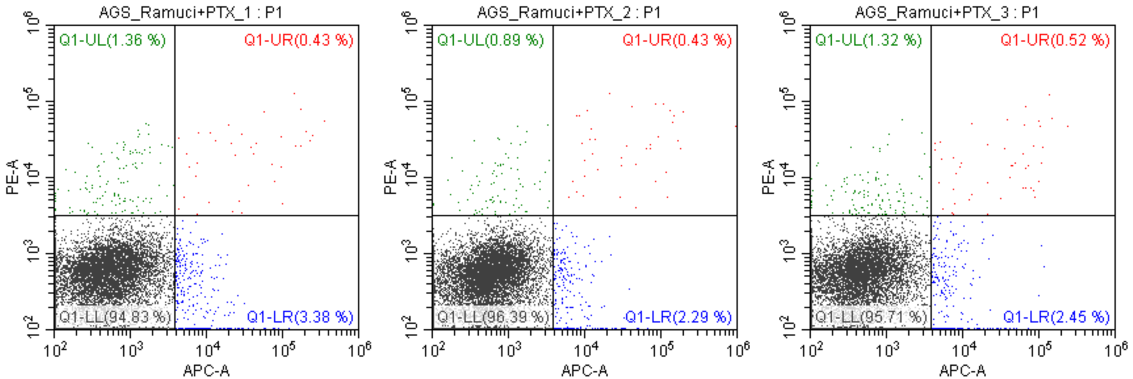

Supplement: Supplementary file 1 [file ijms-25-01769-s001.zip › ijms-2798657-supplementary.pdf]
